# Integrating network analysis and experimental validation to reveal the ferroptosis-associated mechanism of acteoside in the treatment of diabetic nephropathy

**DOI:** 10.3389/fphar.2025.1660227

**Published:** 2025-09-24

**Authors:** Zheng Luo, Shu-Jiao Zhang, Jing-Yi Tang, Yuanyuan Deng, Yi Kang, Xing-Hua Zhang, Hong-Mei Lu, Yao-Xian Wang, Wei-Jing Liu, Yu Ma

**Affiliations:** ^1^ Department of Nephrology, Dongfang Hospital, Beijing University of Chinese Medicine, Beijing, China; ^2^ Dongzhimen Hospital, Beijing University of Chinese Medicine, Beijing, China; ^3^ Key Laboratory of Chinese Internal Medicine of Ministry of Education, Beijing Dongzhimen Hospital, Beijing University of Chinese Medicine, Beijing, China; ^4^ Henan University of Chinese Medicine, Zhengzhou, China; ^5^ Renal Research Institution of Beijing University of Chinese Medicine, Beijing University of Chinese Medicine, Beijing, China

**Keywords:** diabetic nephropathy, Acteoside, ferroptosis, Keap1/Nrf2/HO-1, AGEs/RAGE, oxidative stress, network pharmacology

## Abstract

**Background:**

Acteoside (ACT), a natural phenylethanoid glycoside extracted from the *Rehmannia glutinosa*, has demonstrated renal protective effects against diabetic nephropathy (DN) through its confirmed antioxidant and anti-inflammatory properties. However, the underlying mechanisms by which ACT regulates DN progression via targeting ferroptosis remain to be elucidated.

**Purpose:**

This study aims to elucidate whether ACT ameliorates DN in mice by targeting ferroptosis and to uncover the underlying mechanisms involved.

**Methods:**

This study first utilized network pharmacology approaches, integrating multiple databases and bioinformatics tools, to predict and screen the potential targets and pathways of ACT in DN. To validate the therapeutic efficacy of ACT, a DN model was established in C57BL/6J mice using streptozotocin (STZ). Subsequently, the therapeutic effect of ACT on DN was verified through molecular experiments. Finally, molecular docking was adopted to further verify the binding ability between ACT and key targets.

**Results:**

Network pharmacology analysis identified potential targets of ACT related to DN and revealed that its therapeutic effects may be mediated through the regulation of ferroptosis. *In vivo* experiments demonstrated that ACT exerts significant renoprotective effects by improving renal function and alleviating pathological damage in DN mice. Furthermore, ACT was shown to attenuate oxidative stress by restoring mitochondrial homeostasis, a process closely associated with the regulation of ferroptosis.

**Conclusion:**

In summary, this study provides preclinical evidence that ACT ameliorates DN through ferroptosis inhibition, positioning it as a novel therapeutic candidate for DN treatment.

## Graphical Abstract

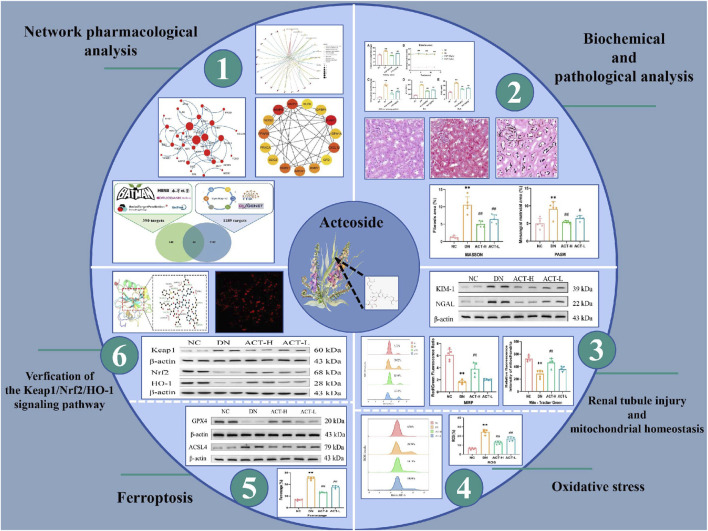

## 1 Introduction

Diabetic nephropathy (DN) is a severe microvascular complication of diabetes mellitus, affecting an estimated 30%–40% of diabetic patients and serving as the leading cause of end-stage renal disease (ESRD) globally (Gheith et al., 2016; Hussain et al., 2021). Clinically, DN is defined by persistent albuminuria and a sustained decline in glomerular filtration rate, with histopathological hallmarks including glomerular basement membrane thickening, mesangial expansion, and tubulointerstitial fibrosis (Hussain et al., 2021). The current treatment strategies recommended by the guidelines mainly include renin-angiotensin system inhibitors (ACE inhibitors or ARBs) and the recently approved sodium-glucose cotransporter-2 inhibitors, which have shown significant efficacy in slowing down the progression of DN. For example, empagliflozin reduced the risk of incident or worsening nephropathy by 39% compared with placebo, and canagliflozin similarly attenuated eGFR decline and albuminuria (Wanner et al., 2024; Neal et al., 2017). Despite these advances, a significant residual risk of renal functional decline and cardiovascular events remains, underscoring the unmet need for novel therapeutic agents that target additional pathogenic pathways (Parving et al., 2016).

Ferroptosis is an iron-dependent form of regulated cell death driven by lipid peroxidation and characterized by the accumulation of iron and reactive oxygen species (Li et al., 2023a). Mounting evidence implicates ferroptosis in DN pathogenesis: hyperglycemia-induced oxidative stress and dysregulated iron metabolism sensitize renal tubular cells to ferroptotic injury, with downregulation of glutathione peroxidase 4 (GPX4) observed in diabetic kidneys (Li L. et al., 2023; Mengstie et al., 2023). Preclinical studies have shown that pharmacological modulation of ferroptosis, for instance via activation of the Nrf2/HO-1 pathway, can attenuate renal injury in DN models (Wang and Chen, 2022). These findings highlight ferroptosis as a critical and potentially druggable pathway in the progression of diabetic kidney damage.

Acteoside (ACT), a natural phenylpropanoid glycoside abundant in Rehmannia glutinosa and other medicinal herbs, possesses potent antioxidant, anti-inflammatory, and antifibrotic properties (Zhou et al., 2024). Recent *in vivo* studies have demonstrated that ACT ameliorates renal interstitial fibrosis and improves kidney function in streptozotocin-induced DN models, reducing serum creatinine and blood urea nitrogen levels while attenuating histopathological damage (Zhou et al., 2024; Gao et al., 2024). However, the precise molecular mechanisms, particularly whether ACT modulates ferroptosis pathways, remain to be elucidated. Integrating network pharmacology with experimental validation offers a powerful strategy to uncover the ferroptosis-associated targets and pathways through which ACT may exert its renoprotective effects in DN.

Network pharmacology enables systematic mapping of bioactive compounds, targets, and signaling pathways, shedding light on the mechanisms by which traditional Chinese medicines act in complex disorders (Li et al., 2023c). In this study, we first applied a network pharmacology framework, integrating compound, target predictions, and pathway enrichment analyses to pinpoint the putative molecular targets and pathways through which ACT may exert renoprotective effects in DN. To substantiate these *in silico* pharmacology predictions, we conducted *in vivo* experiments in STZ-induced DN mice. Finally, molecular docking analyses were performed to validate the direct binding interactions between ACT and the critical ferroptosis-regulatory targets identified.

## 2 Materials and methods

### 2.1 Reagents

RAGE (Biodragon, RM3154, 1:1000), KIM1 (Biodragon, BD-PA1474, 1:1000), NGAL (Biodragon, BD-PT7910, 1:1000), GPX4 (Proteintech, 67763-1-1g, 1:1000), ACSL4 (Proteintech, 22401-1-AP, 1:2000), Keap1 (Biodragon, RM0285, 1:1000), Nrf2 (Proteintech, 16396-1-AP, 1:2000), HO-1 (Proteintech, 10701-1-AP, 1:1000), β-actin (Affinity, AF7018, 1:3000), and GAPDH (Affinity, AF7021, 1:3000). ACT was produced by Yongjian Pharmaceutical Co., Ltd., China. (B01841, purity >98.0% HPLC). Detailed r*eagents* can be found in Supplementary Table S1.

### 2.2 Screening of active components of ACT

ACT’s target prediction utilized four databases: Bioinformatics Analysis Tool for BATMAN-TCM (http://bionet.ncpsb.org.cn/batman-tcm/), DrugBank (https://go.drugbank.com/), HERB (http://herb.ac.cn/), and SwissTargetPrediction (http://www.swisstargetprediction.ch). The predicted targets were imported into the UniProt database (https://www.uniprot.org/) for normalization and duplicate removal, yielding unique target values. Disease-related targets for diabetic nephropathy were identified by querying DisGeNET (https://www.disgenet.org/), SymMap (http://www.symmap.org/), and TTD (http://db.idrblab.net/ttd) and then removing duplicates. A Venn diagram illustrating the overlap between component and disease targets was created using the EVenn online platform.

### 2.3 PPI analysis and core target screening

The overlapping targets were input into the STRING database with a confidence threshold of ≥0.4 to construct a PPI network. The resulting data were retrieved from STRING and imported into Cytoscape to create a visual molecular interaction network. The key hub genes were pinpointed using the MCC algorithm, which was implemented through the CytoHubba plugin in Cytoscape. The top 15 targets with the highest MCC scores were selected as the core targets for ACT in treating DN.

### 2.4 Functional enrichment analysis

Gene functions and pathways were analyzed using enrichment analyses for GO and KEGG with R software. This process employed the “ggplot,” “enrichplot,” and “clusterProfiler” packages, with the significance level set to *p* < 0.05. The outcomes were visualized to facilitate interpretation.

### 2.5 Animal preparation

Fifty healthy male C57BL/6J mice, aged 6–8 weeks and of SPF grade, were sourced from Beijing Wonderful Oasis Biotechnology Co., LTD. (animal license number SYXK (jin) 2019–0001). The mice were kept in an SPF-grade facility at the Experimental Animal Centre, Dongfang Hospital, University of Chinese Medicine, Beijing, China. Before modeling, the mice were acclimatized for 1 week under controlled conditions: a room temperature of 21 °C ± 2 °C, 50%–60% relative humidity, and a 12-h light/dark cycle. During this period, they had a standard diet with unlimited food and tap water access.

The Animal Ethics Committee of the Dongfang Hospital, Beijing University of Chinese Medicine, approved all animal experiments (approval number DFYY202131R). We confirm that all experiments were conducted in accordance with the relevant guidelines and regulations. In addition, our research involving live animals complies with the ARRIVE guidelines.

### 2.6 Establishment of mouse model

After 1 week of adaptive feeding, mice were weighed, numbered, and randomly assigned to either a control group (NC, n = 10) or a model group (M, n = 40). Mice in the NC group were given a standard diet. In contrast, the model group received up to three intraperitoneal injections of freshly prepared STZ (45 mg/kg/day) in sodium citrate buffer, administered every other day. Mice in the NC group received injections of citrate buffer in equivalent volumes. Before each injection, mice were fasted for 12 h overnight but were allowed access to water. Injections were completed within half an hour (Aydın and Nazıroğlu, 2023; Chen et al., 2023). After the modeling phase, mice resumed feeding and underwent another 12-h fast 7 days later. The model was validated when fasting blood glucose levels, measured via tail-tip blood sampling, reached or exceeded 16.7 mmol/L for three consecutive days, including the day of the measurement, serving as an indicator of successful model establishment (Li et al., 2024). The 40 mice with confirmed successful modeling were randomly divided into the DN, ACT-H, and ACT-L groups. Beginning on day 3 after successful modeling, based on previous studies and our preliminary experiments, mice in the ACT group received low and high doses of ACT (40 and 80 mg kg^-1^·d^-1^, ig once daily) for 12 weeks, and both control and model groups were given daily intragastrical gavage of equal amounts of double distilled water (Gao et al., 2024; Li et al., 2023d; Mao et al., 2023) (Supplementary Table S2).

### 2.7 Collection and preparation of sample

After modeling, mice body weights in each group were recorded using an electronic balance every 4 weeks. Before euthanasia, the final body weight was recorded, and the right kidney was excised for weight measurement. Blood glucose levels were checked every 4 weeks via the tail vein using a rapid glucose meter. At week 12, mice were placed in metabolic cages with free access to water and no food, and total urine output was collected over 24 h. After 12 weeks of treatment, mice were anesthetized with a 1% sodium pentobarbital (40 mg/kg) intraperitoneal injection, followed by blood collection via the ocular route. Mice were then euthanized with a second dose of sodium pentobarbital, and both kidneys were harvested and weighed. The right kidney was cut, fixed in 4% paraformaldehyde, dehydrated, embedded in paraffin, and sectioned for HE, MASSON, and PASM staining. The remaining kidney tissue was stored in liquid nitrogen at −80 °C for future analysis.

### 2.8 Detection of biochemical indexes

A glucometer assessed blood glucose levels every 4 weeks through tail vein puncture. The 24-h UTP, serum scr and serum BUN concentrations of mice were detected with the specified detection kit.

### 2.9 Detection of ROS

Renal tissue ROS levels were assessed by incubating collected cells with 10 µM DCFH-DA, achieved by diluting 2′,7′-dichlorodihydrofluorescein diacetate in serum-free medium at a 1:1000 ratio. Cells were incubated at 37 °C in the dark for 20 min, washed with serum-free medium, and analyzed via flow cytometry.

### 2.10 Detection of AGEs and oxidative stress

Serum concentrations of AGEs were measured using the specified assay kits. A sample of renal tissue was excised and processed into a homogenate. The tissue homogenate was centrifuged at 3500 rpm for 10 min at 4 °C, and the supernatant was collected. The concentrations of GSH, SOD, LPO, and MDA in the homogenate were measured using appropriate assay kits, following the manufacturer’s protocols carefully.

### 2.11 MPTP assay and measurement of the MMP

The mitochondrial MPTP opening in renal tissue in mice was detected using the MPTP Assay Kit. After collecting the cells, stain them with the prepared Calcein AM staining solution and observe using a flow cytometer.

Subsequently, to evaluate the MMP in renal tissues, a mitochondrial extraction kit along with the Enhanced MMP Assay Kit using JC-1 was employed for tissue preparation and staining. Fluorescent enzymatic labeling was then applied for detection. The JC-1 monomer was measured with excitation at 490 nm and emission at 530 nm, whereas the JC-1 polymer was assessed with excitation at 525 nm and emission at 590 nm. The fluorescence intensity ratio was calculated to assess the MMP.

### 2.12 Mitochondrial content assay

To assess the mitochondrial content of renal tissues, the cells are collected and then loaded with MitoTracker^®^ Green FM at 37 °C for 30 min to label the mitochondria. Following three PBS washes, fluorescent enzyme labeling was detected at an excitation wavelength of 490 nm and an emission wavelength of 530 nm.

### 2.13 Detection of iron concentration in serum and renal tissue

Iron levels in serum and renal tissue were measured using commercially available assay kits, following the manufacturer’s instructions. Furthermore, the iron concentration in renal tissue was assessed using FerroOrange. The working fluid was pre-prepared, following the provided guidelines. Following cell collection, a 1 μmol/L solution was introduced, and the cells were incubated for 30 min before analysis with a flow cytometer.

### 2.14 Western blot (WB)

WB analysis was performed to evaluate the expression of RAGE, KIM-1, NGAL, GPX4, ACSL4, Keap1, Nrf2, HO-1, β-actin, and GAPDH in renal tissue. Proteins were extracted using lysis buffer, quantified, and separated by electrophoresis. A total of 20 μg of protein from each sample was transferred to a nitrocellulose membrane, which was blocked with 5% skim milk. The membrane was incubated with primary and secondary antibodies, and protein bands were detected using an enhanced chemiluminescence kit and visualized with a ChemiScope system. Band intensities were quantified using ImageJ software.

### 2.15 RT-qPCR

To extract RNA from renal tissue, follow the instructions provided in the reagent manual. Subsequently, convert the extracted RNA into cDNA following the reverse transcription kit instructions. Prepare a 20 µL reaction mixture as detailed in Supplementary Table S3. The reaction procedure is shown in the Supplementary Table S4. The 2^−ΔΔCT^ method, with β-actin as the internal reference, calculates the mRNA expression levels of Keap1, Nrf2, and HO-1. The primer sequences can be found in Supplementary Table S5.

### 2.16 Immunofluorescence (IF) staining

Renal tissue sections were incubated overnight with antibodies targeting RAGE, Keap1, and Nrf2, each at a dilution of 1:50. Following PBS washing, sections were incubated with goat anti-rabbit secondary antibodies for 1 h at 4 °C, followed by nuclear staining with 4′,6-diamidino-2-phenylindole (DAPI). Colocalization of RAGE, Keap1, and Nrf-2 was evaluated with a fluorescence microscope.

### 2.17 Molecular docking validation

Initially, we obtained three-dimensional structures of target genes, functioning as protein receptors, from the PDB database (http://www.rcsb.org/). ACT chemical compounds were sourced as ligands from the PubChem database (https://pubchem.ncbi.nlm.nih.gov/). Using the AutoDock Vina docking model, we then calculated the affinity between these receptors and ligands. An affinity value of ≤ −5.0 kcal/mol typically signifies a strong interaction between the receptor and ligand (Li et al., 2023c). A lower ligand-receptor affinity indicates more stable binding. Finally, the outcomes were visualized using PyMOL v2.3.0 and LigPlot v2.2.1 software for enhanced comprehension.

### 2.18 Statistical analysis

Data are expressed as mean ± SD, with each experiment performed at least three times. For comparisons between two groups, Student’s t-test was used, while one-way ANOVA with Tukey’s *post hoc* test was applied for multiple groups. Non-parametric data were analyzed using the Wilcoxon signed-rank test. Statistical analysis and graphing were performed using GraphPad Prism 8.0, with a P-value <0.05 considered significant.

## 3 Result

### 3.1 Acquisition of ACT targets

**FIGURE 1:**
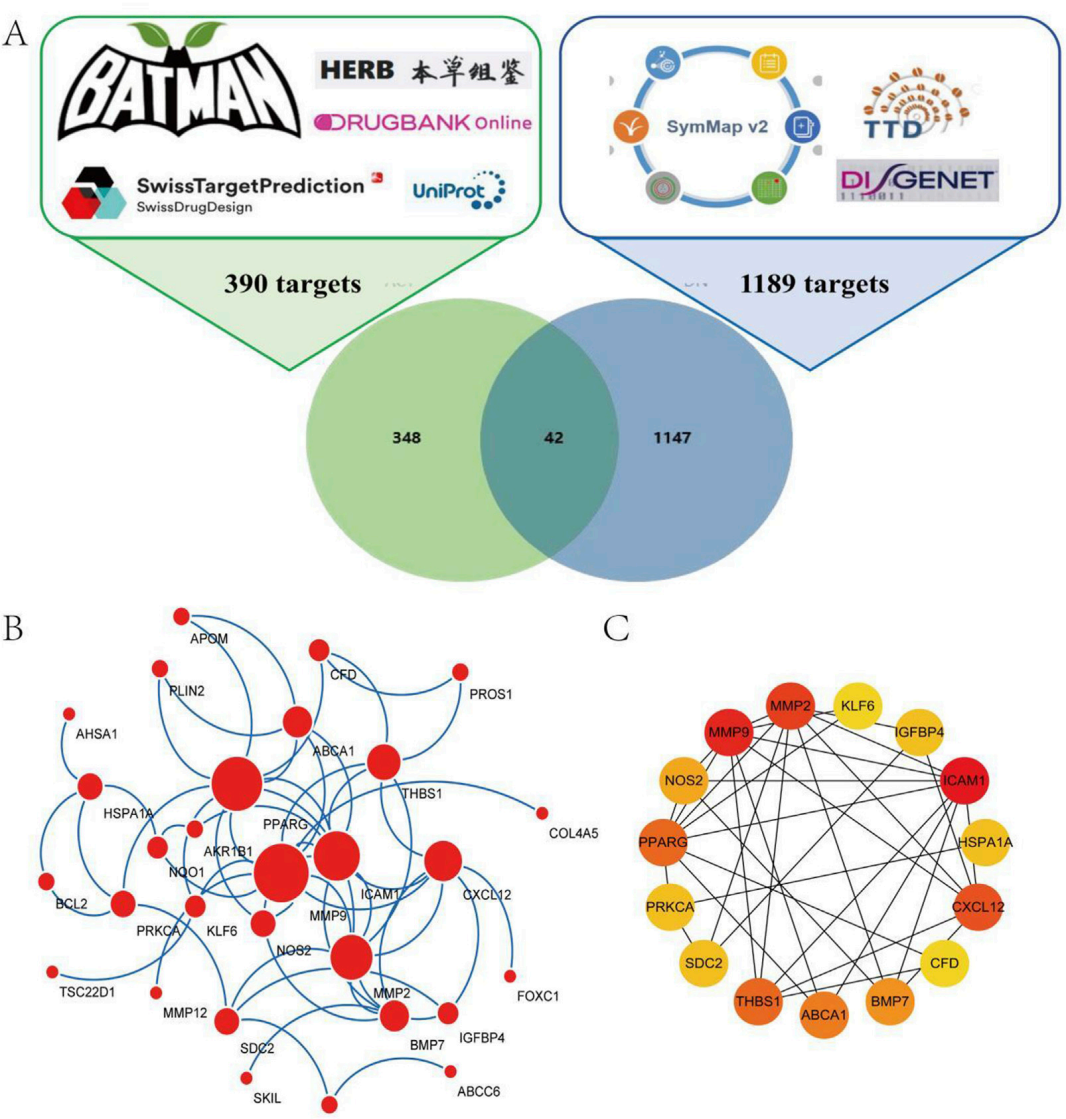
Network analysis of ACT-treated DN. **(A)** 390 targets from ACT, 1189 targets from DN, and a Venn diagram of 42 shared targets of ACT and DN. **(B)** Acquirement of the 33 key targets from the 42 common targets via STRING network topological analysis. **(C)** 15 PPI Key targets obtained through the MCC algorithm of the Cytohubba plugin.

In order to clarify the ACT’s targets, using Batman, DrugBank, HERB, and the Swiss Target Prediction databases for target prediction of the ACT. Summarize the targets by taking union sets. 390 targets were ultimately obtained (Figure 1A) (Supplementary Table S6). A total of 1189 therapeutic targets for DN treatment were identified from DisGeNET, SymMap, and TTD (Figure 1A) (Supplementary Table S7). A Venn diagram was created to identify potential ACT targets in DN. Ultimately, 42 shared targets were identified as potential targets of ACT in the treatment of DN (Figure 1A) (Supplementary Table S8).

### 3.2 PPI network analysis

To assess the significance of potential targets, 42 common targets were uploaded to the STRING database for analysis, excluding those without interaction data. This resulted in 33 targets for topological analysis, leading to the construction of a PPI network (Figure 1B) with 33 nodes and 64 edges, an average node degree of 3.88. The MCC method in the Cytohubba plugin was used to identify the top 15 key targets, including MMP9, BCL2, ICAM1, CXCL12, MMP2, and PPARG (Figure 1C). These targets are primarily associated with oxidative stress, apoptosis, and inflammation.

### 3.3 Functional enrichment analysis

**FIGURE 2:**
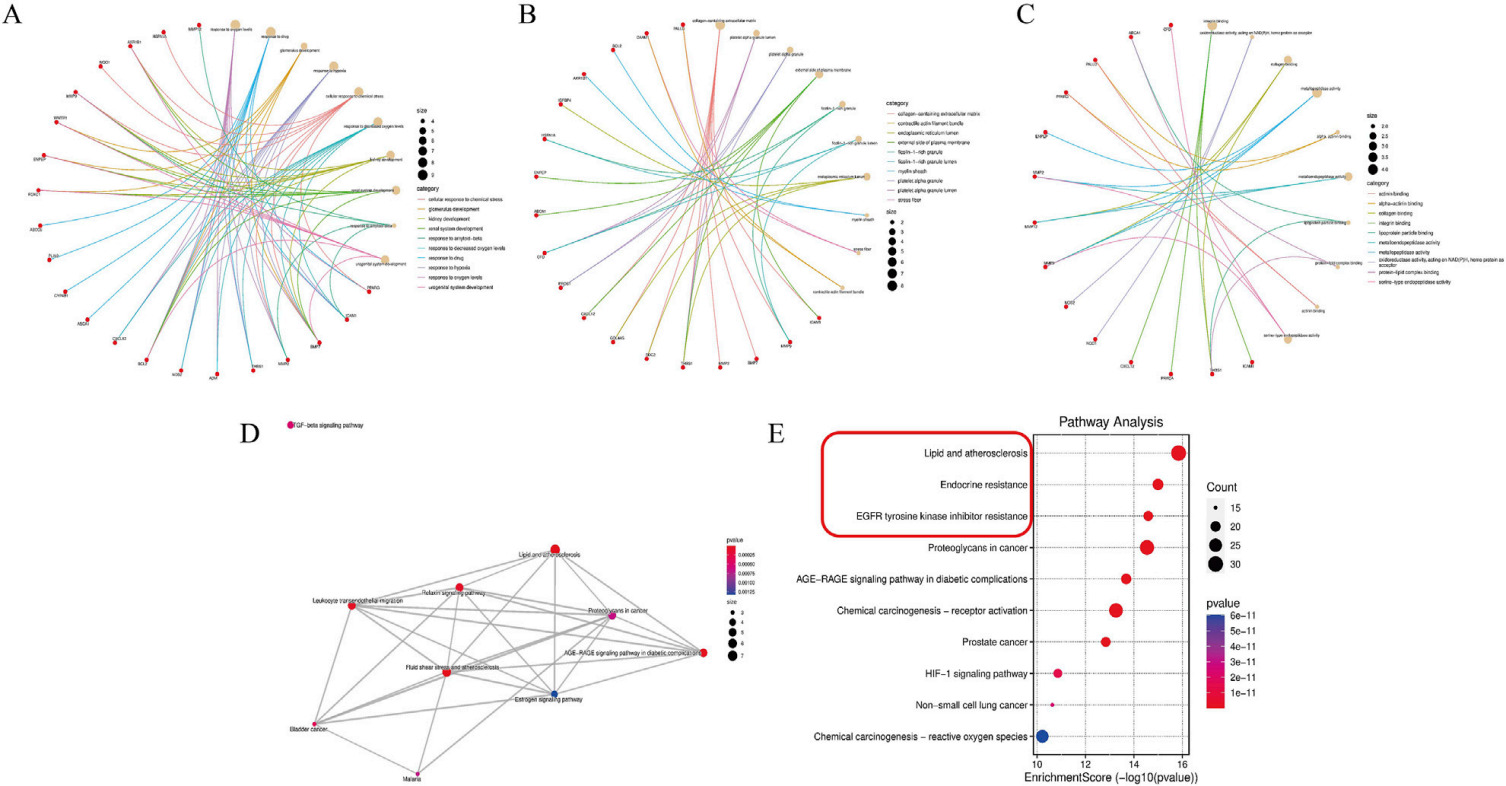
Enrichment analysis of the key targets. **(A–C)** The top 10 significantly enriched terms in biological processes, molecular functions, and cellular components. **(D,E)** The top 10 significantly enriched terms in KEGG pathways.

To explore the functions of the 42 key targets, gene annotation and functional enrichment analyses were conducted (Supplementary Table S9). The top 10 GBP were mainly related to DN treatment, including oxygen level response, drug response, glomerulus development, and hypoxia response (Figure 2A). The leading GMF involved integrin binding, oxidoreductase activity, collagen binding, and metallopeptidase activity (Figure 2B). The top 10 GCC included the collagen-containing extracellular matrix, platelet alpha granule lumen, and external plasma membrane (Figure 2C). KEGG pathway enrichment revealed that ACT’s effects on DN were linked to oxidative stress-related signaling pathways and ferroptosis-related signaling pathways (such as lipid and atherosclerosis, fluid shear stress and atherosclerosis, and the AGE-RAGE signaling pathway in diabetic complications) (Figures 2D,E). These findings are supported by network pharmacology and literature. The key mechanism of ACT in treating DN may be related to alleviating oxidative stress and regulating ferroptosis.

### 3.4 The effect of ACT on general biochemical indicators in DN mice

**FIGURE 3:**
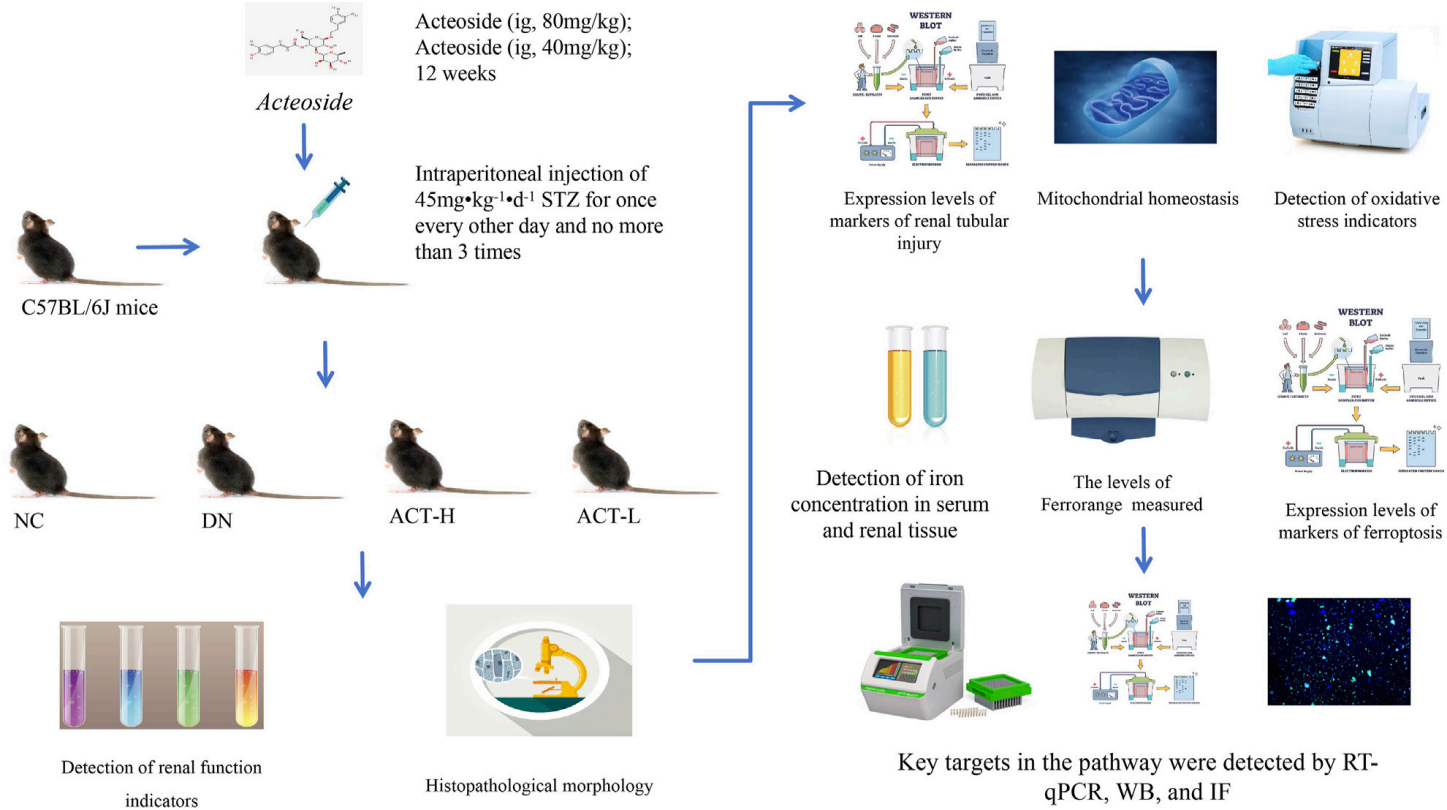
Experimental technology roadmap.

**FIGURE 4:**
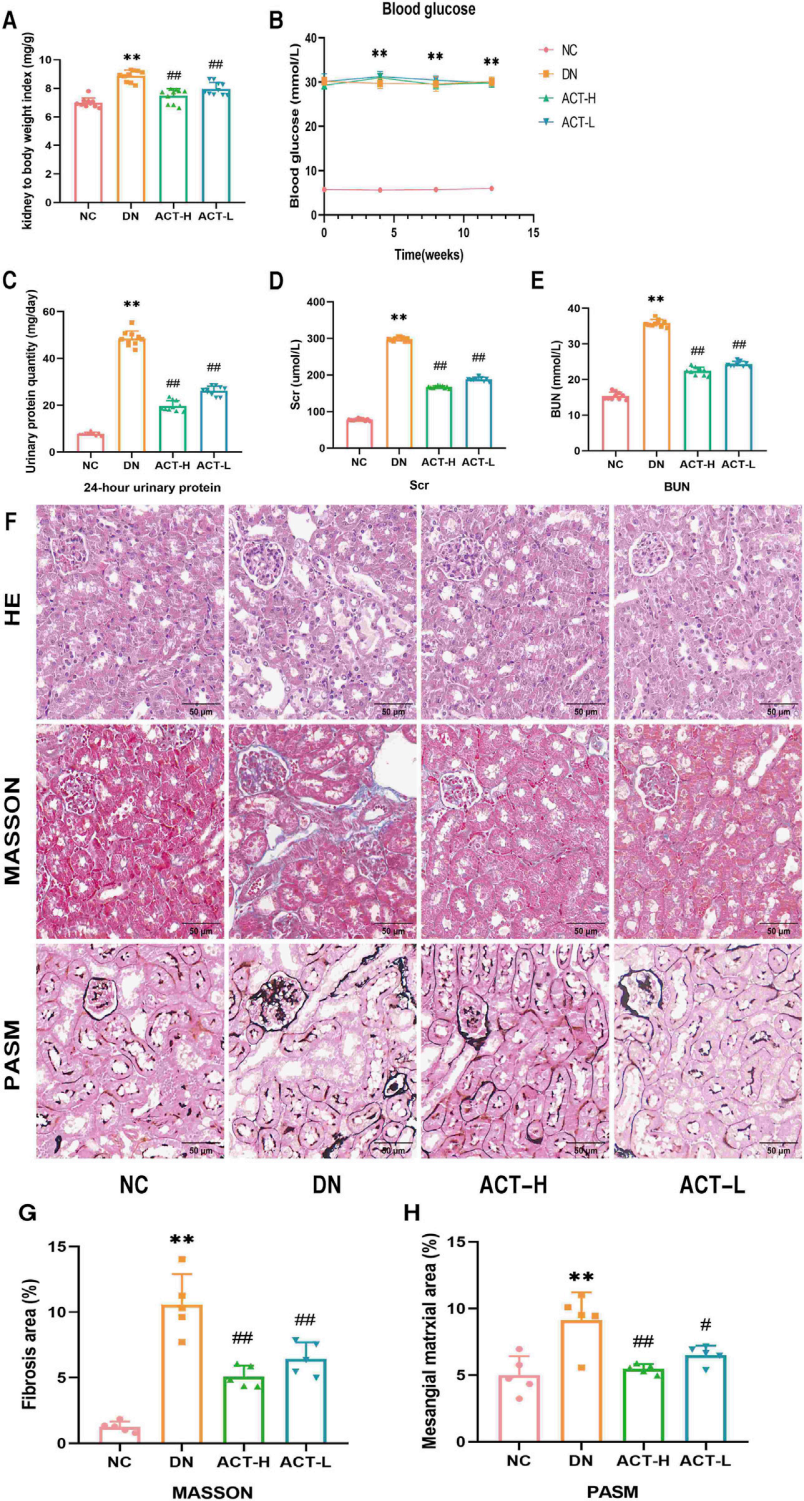
Effects of ACT on DN mice. **(A)** Kidney index. **(B)** Blood glucose. **(C)** 24-h UTP. **(D)** Scr. **(E)** BUN. **(F)** HE, Masson, and PASM staining (200x; scale bar = 50 um). **(G,H)** Bar graph of relative Masson and PASM. **P < 0.01 vs. NC group; ^##^ *P* < 0.05 or ^##^ *P* < 0.01 vs. DN group.

The experimental protocol is illustrated in Figure 3. As shown in Figures 4A–E, in comparison to the NC group, the DN group mice exhibited significant increases in the kidney index, blood glucose, 24-h UTP, Scr, and BUN (*P* < 0.01). Following 12 weeks of drug intervention, the ACT-H and ACT-L groups showed significant reductions in 24-h UTP, Scr, and BUN compared to the DN group (*P* < 0.01 or *P* < 0.01), while blood glucose levels remained statistically unchanged across the groups (Supplementary Table S10).

### 3.5 Effect of ACT on renal histopathological morphology in the DN mice

Renal pathological staining in the DN group mice revealed renal tubular lumen expansion, vacuolation, degeneration, and desquamation of renal tubular epithelial cells, blue collagen fiber deposition, and thickening of the glomerular basement membrane. Following drug intervention, these pathological changes were significantly alleviated in the ACT-H and ACT-L groups compared to the DN group (*P* < 0.05 or *P* < 0.01), as shown in Figures 4F–H (Supplementary Table S10).

### 3.6 ACT rescues renal tubule injury and mitochondrial homeostasis in DN mice

**FIGURE 5:**
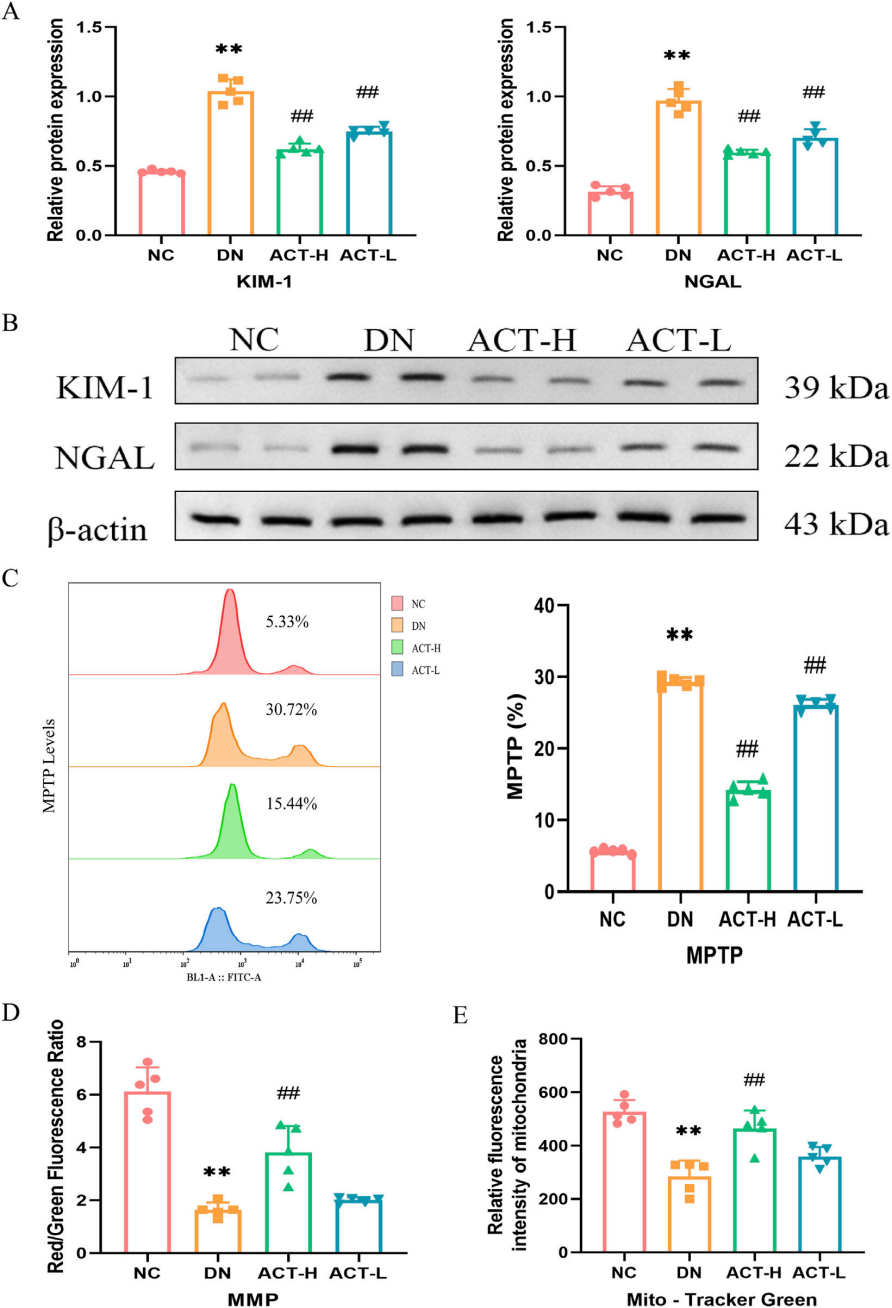
Effects of ACT on renal tubule and mitochondrial homeostasis in DN mice. **(A)** Bar graphs of the relative expressions of KIM-1 and NGAL. **(B)** WB images of the expressions of KIM-1 and NGAL. **(C)** The levels of MPTP production are measured by a flow cytometer. **(D)** The fluorescence intensity of cells labeled by JC-1 was measured by the microplate reader. **(E)** The fluorescence intensity of mitochondria labeled by Mito-Tracker Green was measured by the microplate reader. **P < 0.01 vs. NC group; ^##^ *P* < 0.01 vs. DN group.

Given the critical role of tubular epithelial cell injury in the pathogenesis of DN, we assessed the expression levels of renal injury-specific biomarkers, KIM-1 and NGAL, by WB analysis. As shown in Figures 5A,B, the expression of KIM-1 and NGAL proteins in renal tissues was significantly elevated in the DN group compared with the NC group (*P* < 0.01). Notably, treatment with ACT resulted in a reduction in the expression of these markers (*P* < 0.01), suggesting that ACT may exert renoprotective effects by alleviating tubular injury. In addition, our network pharmacology analysis indicated a strong association between ACT’s therapeutic effects and oxidative stress regulation. Since mitochondria are major sources of ROS and mitochondrial homeostasis is closely linked to tubular damage, we systematically evaluated the regulatory effects of ACT on mitochondrial function. First, we assessed the opening state of the MPTP using Calcein AM fluorescent staining. As illustrated in Figure 5C, DN mice showed a significant reduction in fluorescence intensity (*P* < 0.01), indicative of excessive MPTP opening, whereas ACT treatment significantly restored fluorescence levels (*P* < 0.01), implying effective inhibition of MPTP opening by ACT. Furthermore, MMP was evaluated using JC-1 dye. As shown in Figure 5D, the red/green fluorescence ratio was significantly decreased in the DN group compared to the NC group (*P* < 0.01), while ACT-H treatment reversed this decline (*P* < 0.01). Mitochondrial morphological analysis (Figure 5E) revealed apparent mitochondrial fragmentation in the DN group, whereas mitochondrial density and structural integrity were restored following ACT treatment (*P* < 0.01). These findings suggest that ACT restores impaired mitochondrial homeostasis in DN through multiple mechanisms, including inhibition of MPTP opening, stabilization of MMP, and preservation of mitochondrial network integrity. Collectively, these results indicate that ACT may confer renoprotective effects by mitigating tubular epithelial cell injury and restoring mitochondrial quality control, thereby slowing the progression of DN (Supplementary Table S11).

### 3.7 ACT alleviated the levels of oxidative stress in the DN mice

**FIGURE 6:**
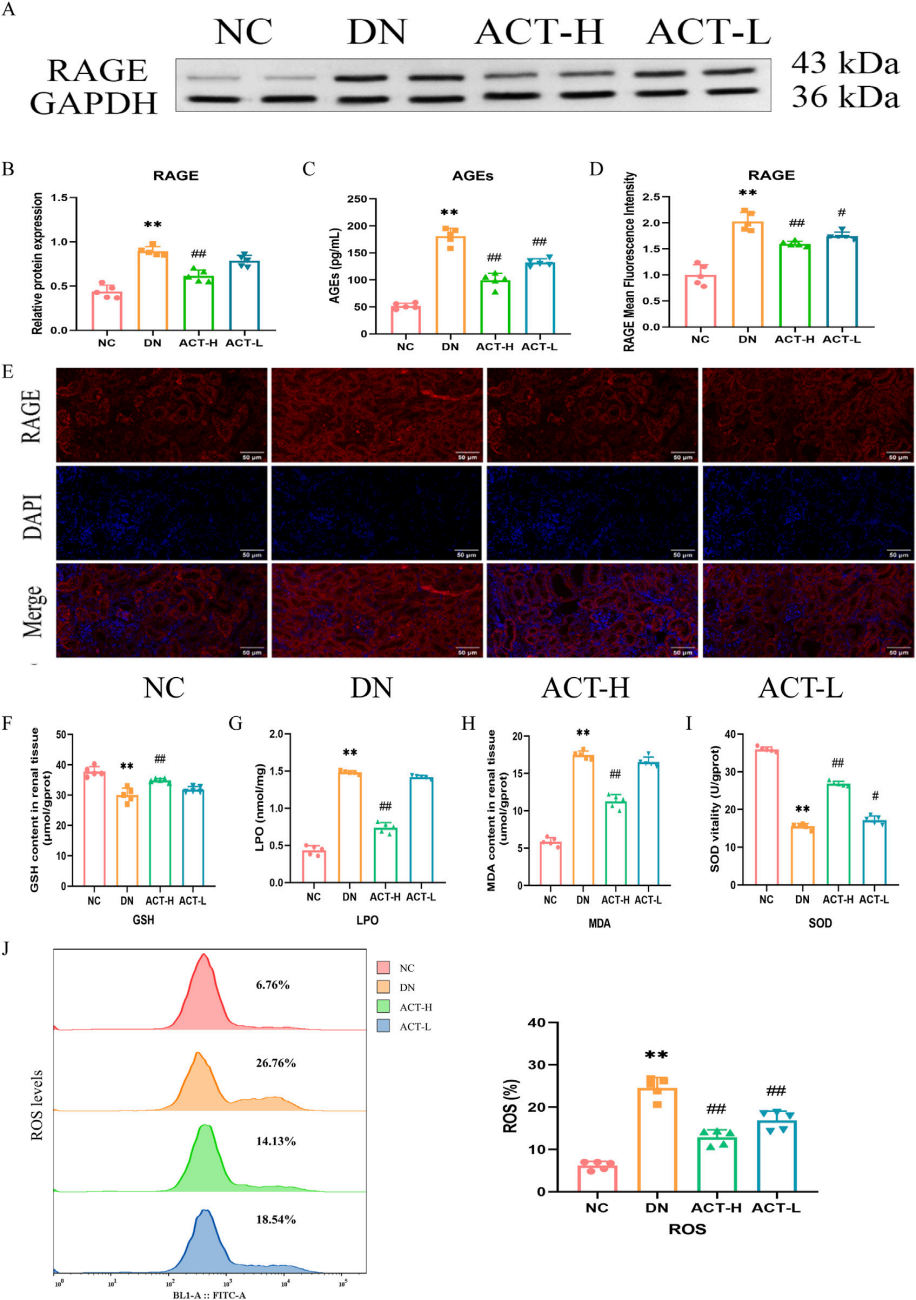
Effects of ACT on oxidative stress in the DN mice. **(A)** WB images of the expressions of RAGE. **(B)** Bar graphs of the relative expressions of RAGE. **(C)** Bar graphs of the levels of AGEs. **(D)** The graphs show quantitation data of an average of 5 independent RAGE IF stainings. **(E)** IF analysis that shows the abundance and distribution of RAGE. **(F–I)** Bar graphs of the levels of GSH, LPO, MDA, and SOD. **(J)** The levels of ROS production measured by flow cytometer in each group. Representative micrographs were given (200 x; scale bar = 50 um). **P < 0.01 vs. NC group; ^#^ *P* < 0.01 or ^##^ *P* < 0.01 vs. DN group.

Mitochondrial dysfunction is closely related to oxidative stress. Network analysis and current evidence suggest that ACT may ameliorate DN by modulating oxidative stress via the AGEs/RAGE signaling pathway. We measured serum AGEs and RAGE expression. ELISA revealed elevated AGEs in DN mice (*P* < 0.01) that were reduced by ACT-H intervention. WB and IF confirmed RAGE upregulation in the DN group (*P* < 0.01) and its significant downregulation following ACT-H treatment (Figures 6A–E). Furthermore, we systematically profiled dynamic changes in oxidative-stress biomarkers. In DN mice, serum levels of LPO and MDA were significantly elevated (*P* < 0.01), while SOD activity and GSH content were markedly reduced compared with normal controls. ACT-H treatment effectively reversed these alterations, lowering LPO and MDA and restoring SOD and GSH toward baseline levels (Figures 6F–I; Supplementary Table S12). Recognizing ROS as central mediators of oxidative injury, we quantified ROS generation in renal tissue by DCFH-DA staining and flow cytometry. The DN group exhibited a pronounced increase in ROS fluorescence intensity vs. the NC group (*P* < 0.01), which was significantly attenuated by ACT-H (Figure 6J; *P* < 0.01). Together, these data demonstrate that ACT mitigates oxidative stress in DN by suppressing ROS and lipid peroxidation, enhancing antioxidant defenses (GSH, SOD), and inhibiting the pro-oxidant AGEs/RAGE signaling pathway.

### 3.8 ACT inhibits ferroptosis in DN mice

**FIGURE 7:**
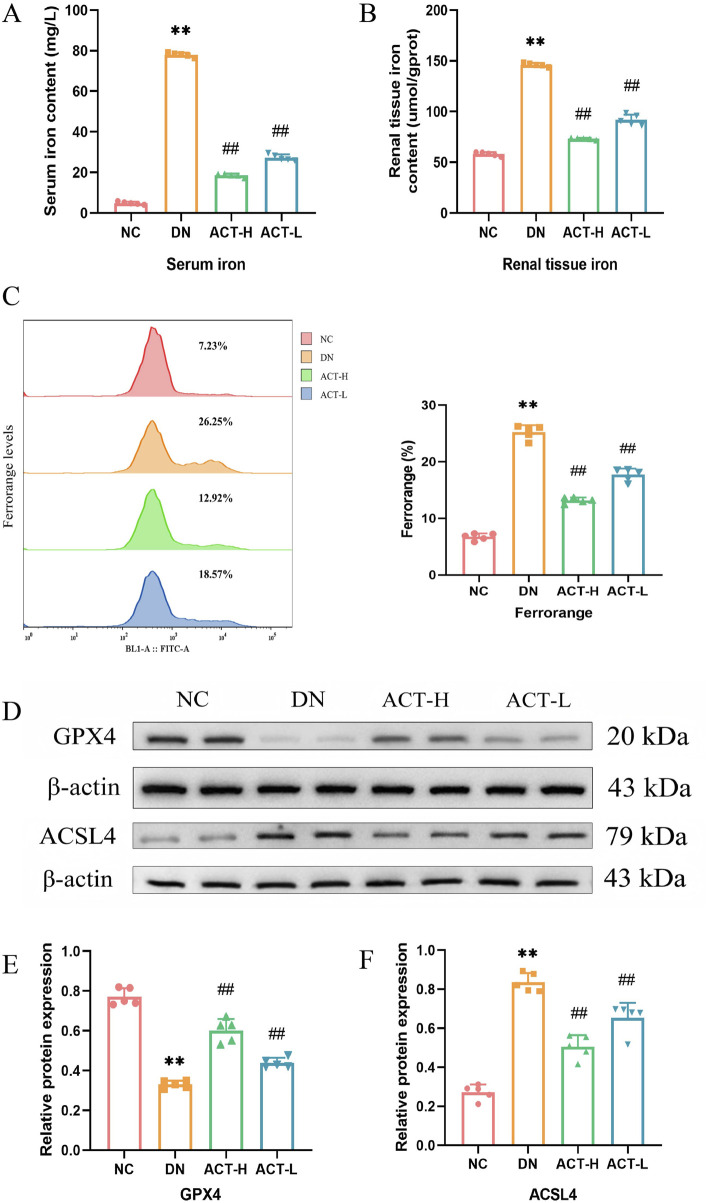
Effects of ACT on ferroptosis of DN mice. **(A)** Serum iron concentration in each group of mice. **(B)** Iron content in renal tissue of mice in each group. **(C)** Representative pictures of Mito Tracker Green fluorescent cells. **(D)** WB images of the expressions of GPX4 and ACSL4. **(E,F)** Bar graphs of the relative expressions of GPX4 and ACSL4. **P < 0.01 vs. NC group; ^##^ *P* < 0.01 vs. DN group.

Given the potential pathological mechanism by which the synergistic effects of mitochondrial homeostasis imbalance and oxidative stress may trigger ferroptosis, we next examined its impact on ferroptosis, an iron-dependent form of programmed cell death characterized by iron accumulation and lipid peroxidation driven by ROS (Yan et al., 2021). Mounting evidence implicates ferroptosis in DN, where hyperglycemia-induced iron overload exacerbates ROS production and tubular cell damage. To assess ACT’s ability to attenuate iron overload, we measured intracellular iron levels in serum and renal tissue. In DN mice, both serum and renal iron concentrations were significantly elevated compared with normal controls (*P* < 0.01; Figures 7A,B), whereas ACT treatment significantly reduced these levels (*P* < 0.05 or *P* < 0.01). Flow cytometric analysis using an iron-sensitive fluorescent probe further confirmed that ACT markedly decreased intracellular iron fluorescence intensity (Figure 7C). To validate the molecular hallmarks of ferroptosis, we evaluated the expression of GPX4 and ACSL4, core regulators of the ferroptotic cascade. In the DN group, GPX4 was downregulated while ACSL4 was upregulated, consistent with enhanced ferroptotic susceptibility. ACT treatment reversed these expression changes, restoring GPX4 levels and suppressing ACSL4 expression (Figures 7D–F; Supplementary Table S13). Collectively, these findings indicate that ACT mitigates ferroptosis in DN by reducing iron overload and normalizing critical ferroptosis regulators, thereby complementing its renal-protective effects.

### 3.9 ACT suppressed activation of the Keap1/Nrf2/HO-1 signaling pathway in the DN model

**FIGURE 8:**
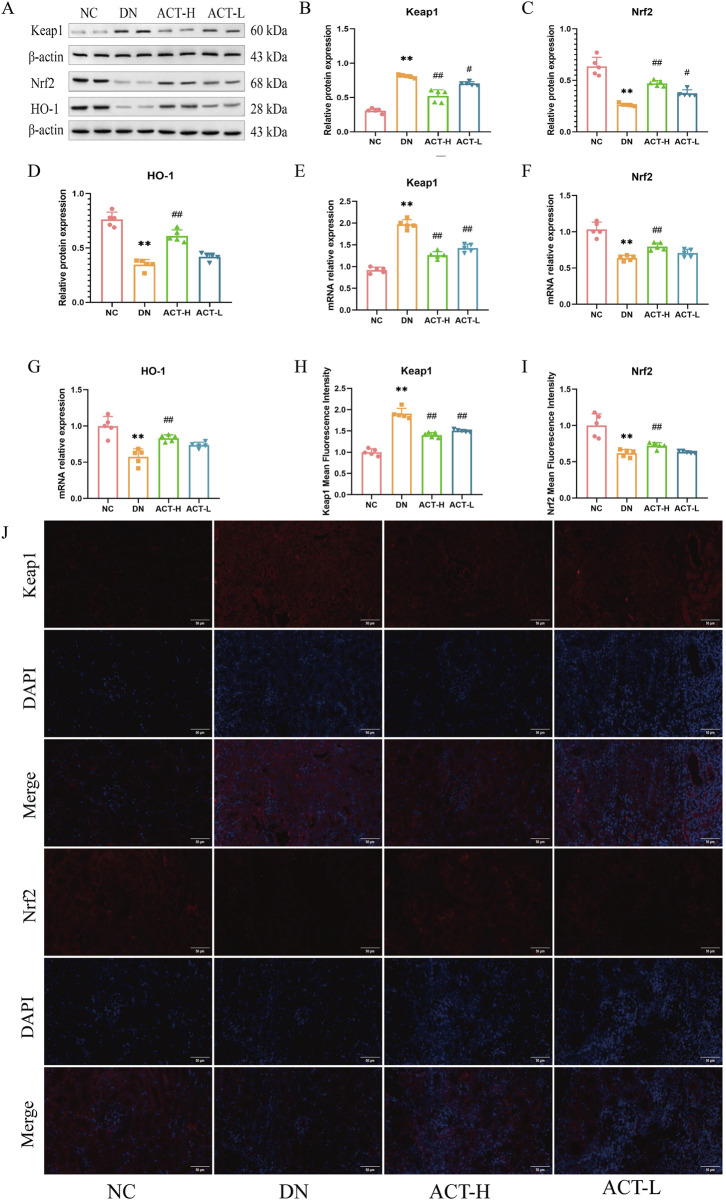
Effects of ACT on keap1, Nrf2, and HO-1 expression of renal in DN mice. **(A)** The effect of ACT on Keap1, Nrf2, and HO-1 protein expression was investigated by WB. **(B–D)** Statistical analysis of the Keap1, Nrf2, and HO-1 protein bands. **(E–G)** The effect of ACT on Keap1, Nrf2, and HO-1 mRNA expression was investigated by RT-PCR. **(H,I)** The graphs show quantitation data of an average of 5 independent Keap1 and Nrf2 IF stainings. **(J)** IF analysis that shows the abundance and distribution of Keap1 and Nrf2. Representative micrographs were given (200 x; scale bar = 50 um). **p < 0.01 vs. NC group; ^#^ *p* < 0.05 vs. DN group. **P < 0.01 vs. NC group; ^#^ *P* < 0.01 or ^##^ *P* < 0.01 vs. DN group.

Given the dynamic interplay between redox imbalance and cellular defense systems during ferroptosis, this study focused on the pivotal role of the Keap1/Nrf2/HO-1 signaling axis in the renoprotective effects mediated by ACT. As a key cellular defense mechanism against ferroptosis, this pathway regulates the expression of various antioxidant and anti-ferroptotic genes through Nrf2 nuclear translocation. To evaluate its involvement, we assessed the expression levels of Keap1, Nrf2, and HO-1 in renal tissues of DN mice treated with ACT using RT-qPCR, WB, and IF. WB results revealed a significant upregulation of Keap1 protein in DN kidneys compared to controls, which was reversed by ACT treatment (*P* < 0.01). In contrast, Nrf2 and HO-1 protein levels were markedly suppressed in DN but significantly restored following ACT intervention (*P* < 0.01) (Figures 8A–D). These protein changes were corroborated by mRNA expression data and IF staining, indicating robust activation of the Keap1/Nrf2/HO-1 axis by ACT. Collectively, these findings suggest that ACT alleviates ferroptosis in DN by preventing Keap1-mediated degradation of Nrf2 and enhancing HO-1-driven antioxidant defenses (Figures 8E–J; Supplementary Table S14).

### 3.10 Molecular docking analysis

**FIGURE 9:**
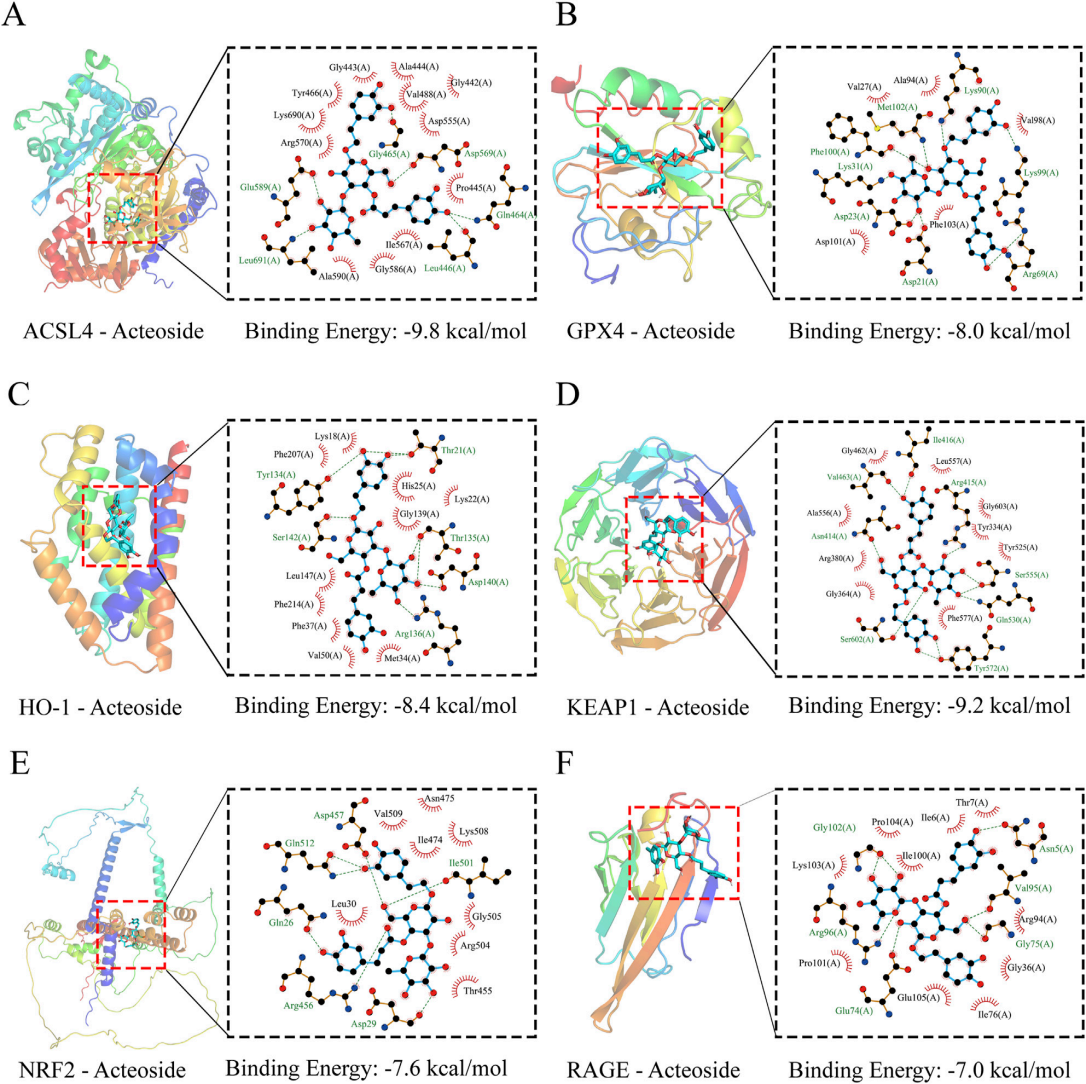
Molecular docking results. **(A–F)** Three-dimensional (3D) and two-dimensional (2D) structural diagrams of ASCL4, GPX4, HO-1, Keap1, Nrf2, and RAGB interacting with ACT, respectively.

Molecular docking was utilized to assess the affinity of identified ACT components towards key targets. The docking binding energies of ACSL4, GPX4, RAGE, HO-1, Keap1, and Nrf2 proteins with ACT are −9.8, −8.0, −8.4, −9.2, −7.6, and −7.0 kcal mol^-1^, respectively, all below −5 kcal mol^-1^, indicating strong binding affinity. The detailed docking conditions are shown in Supplementary Table S15. As shown in Figures 9A–F, it was suggested that ACT predominantly binds to the key targets mentioned, demonstrating a strong binding affinity.

## 4 Discussion

DN represents a multifactorial disorder in which hyperglycemia-induced iron overload and reactive ROS accumulation within renal tubular cells drive progressive nephron injury and fibrosis (Li L. et al., 2023; Chu et al., 2024). Under diabetic conditions, excessive labile iron catalyzes Fenton chemistry, amplifying lipid peroxidation and cellular damage, while mitochondrial dysfunction further exacerbates ROS generation, creating a vicious cycle of oxidative injury (Geng et al., 2024; Jin et al., 2023). Maintenance of both iron and redox homeostasis is therefore essential for preserving tubular epithelial integrity and glomerular function (Chu et al., 2024). Although preclinical studies targeting ferroptosis have demonstrated renoprotective effects in DN models, translation to clinical application remains limited by incomplete mechanistic understanding and off-target toxicity (Li et al., 2025; Daehn et al., 2023). ACT, a phenylpropanoid glycoside with established antioxidant, anti-inflammatory, and antifibrotic properties, has shown promise in ameliorating renal dysfunction in streptozotocin-induced DN mice (Jin et al., 2023). However, its potential to modulate ferroptosis was previously unexplored. Here, by integrating network pharmacology with *in vivo* validation and molecular docking, we demonstrated that ACT restores iron and redox balance, inhibits lipid peroxidation, and activates the AGEs/RAGE and Keap1/Nrf2/HO-1 axis in DN (Miao et al., 2023).

Our network pharmacology analysis identified 42 overlapping targets of ACT and DN by integrating data from BATMAN, DrugBank, HERB, SwissTargetPrediction, DisGeNET, SymMap, and TTD. Mapping these targets onto STRING yielded a PPI network of 33 nodes and 64 edges, from which the top 15 hub proteins (including MMP9, BCL2, ICAM1, CXCL12, MMP2, and PPARG) were highlighted using the MCC algorithm in CytoHubba. The GO and KEGG enrichment of the full target set revealed strong associations with oxidative-stress response, lipid metabolism, and AGE-RAGE signaling. These pathways are intimately connected to ferroptosis: oxidative stress and lipid peroxidation are core initiators of ferroptotic cell death, while disruptions in lipid and iron metabolism prime cells for ferroptosis (e.g., GPX4 inactivation, ACSL4-mediated lipid remodeling) (Wu and Huang, 2024; Yang et al., 2023). Additionally, activation of the AGE-RAGE axis is known to stimulate NADPH oxidase-mediated ROS generation and suppress antioxidant defenses, thereby creating a permissive environment for ferroptosis (Wang and Zhang, 2024; Chen et al., 2024a). Although ferroptosis was not directly annotated as a top enriched pathway, multiple ferroptosis-related processes were, including abnormal lipid metabolism and ROS accumulation. Taken together, these network pharmacology insights suggest that ACT’s core therapeutic mechanism in DN may converge on modulating ferroptosis, providing a rational basis for subsequent experimental validation.

In order to further explore the potential mechanism, we conducted *in vivo* experiments to validate the results of the network analysis above. Our STZ-induced DN mice model, established through multiple low-dose injections of streptozotocin, reliably recapitulated key features of human DN, including persistent hyperglycemia, renal hypertrophy, and impaired renal function (Tesch and Allen, 2007; Zhu et al., 2020). Such biochemical alterations are hallmark indicators of nephron loss and glomerular barrier dysfunction in rodent models of DN (Alaofi, 2020). After 12 weeks of ACT treatment, both low-and high-dose groups exhibited significant reductions in Scr, BUN, and UTP compared with untreated DN mice, while blood glucose remained unchanged, indicating that ACT’s renoprotective effects are independent of glycemic control. Histopathological assessment further revealed pronounced attenuation of tubular lumen dilation, epithelial vacuolation, collagen deposition, and glomerular basement membrane thickening following ACT administration (Alaofi, 2020; Ni et al., 2016). These structural improvements align with ACT’s known antifibrotic properties, as previously reported in other renal injury models (Ma et al., 2023). Together, these data confirm that ACT effectively preserves both renal function and tissue architecture in DN.

Building on the restoration of renal function and tissue architecture, it is essential to consider the broader significance of tubular epithelial integrity in DN. Tubular injury markers (KIM-1 and NGAL) have emerged as sensitive predictors of tubular damage and chronic kidney disease progression in diabetes, correlating with interstitial fibrosis and eGFR decline (Quang et al., 2020). Interventions that reduce KIM-1 and NGAL expression have demonstrated robust renoprotective effects, underscoring the translational relevance of our observations with ACT.

It is crucial to address oxidative stress as a central driver of DN progression. Elevated LPO and MDA levels, alongside diminished SOD activity and GSH content, serve as robust indicators of redox imbalance in DN and correlate with disease severity and rate of eGFR decline (Darenskaya et al., 2023). Excessive ROS production not only damages cellular macromolecules but also activates profibrotic and pro-inflammatory pathways, exacerbating glomerulosclerosis and tubulointerstitial fibrosis. Importantly, the AGEs/RAGE axis perpetuates oxidative stress by stimulating mitochondrial ROS generation, thereby creating a self-amplifying cycle of injury (Wu et al., 2021; Taguchi and Fukami, 2023). Therapeutic strategies that bolster endogenous antioxidant defenses have shown promise in preclinical DN models, reducing albuminuria and histopathological damage (S et al., 2024). Our demonstration that high-dose ACT normalizes LPO, MDA, SOD, and GSH levels, attenuates ROS accumulation, and downregulates AGEs/RAGE signaling thus aligns with these paradigms and underscores the multifaceted antioxidant capacity of ACT.

Building on our demonstration that ACT mitigates oxidative stress, we turned our attention to ferroptosis that sits at the nexus of redox imbalance and mitochondrial dysfunction (Li et al., 2023e). In fact, disruption of mitochondrial homeostasis is a hallmark of ferroptosis, as impaired mitochondrial membrane potential and excessive opening of the permeability transition pore exacerbate lipid peroxidation and ROS generation (Forbes and Thorburn, 2018; Li and Susztak, 2025). In DN, excessive labile iron pools drive ROS generation via Fenton reactions and trigger a vicious cycle of mitochondrial damage and lipid peroxide accumulation, ultimately compromising tubular epithelial integrity (Wang et al., 2023; Chen et al., 2024b). Central to ferroptosis is the depletion of GPX4, which under normal conditions reduces lipid hydroperoxides and safeguards membrane integrity; its downregulation is both a marker and driver of ferroptotic cell death (Yan et al., 2023). Concurrently, ACSL4 facilitates the incorporation of polyunsaturated fatty acids into phospholipids, rendering membranes susceptible to peroxidation (Zhang et al., 2024). Our finding that ACT restores GPX4 levels and suppresses ACSL4 expression aligns with preclinical studies demonstrating that it not only interrupts iron- and ROS-mediated lipid damage but also stabilizes mitochondrial function, thereby attenuating DN injury (Wang and Chen, 2022).

**FIGURE 10:**
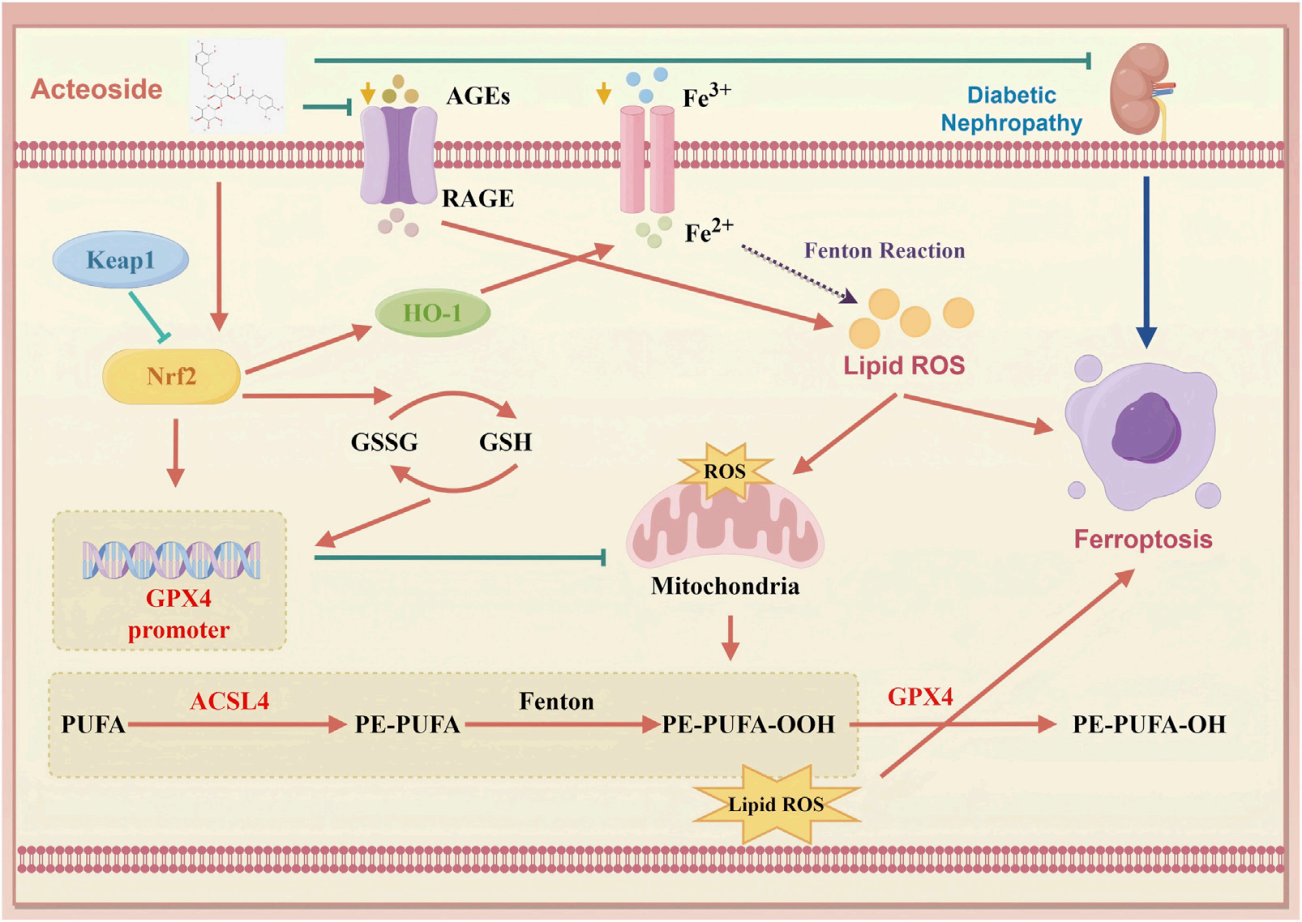
Schematic of ACT’s therapeutic mechanism in DN. ACT protects the renal by reducing oxidative stress and inhibiting ferroptosis via suppression of the AGEs/RAGE and Keap1/Nrf2/HO-1 signaling pathways. (created with www.home-for-researchers.com, ID: WYOOU11911).

The Keap1/Nrf2/HO-1 axis serves as a master regulator of antioxidant and anti‐ferroptotic defenses, where oxidative or electrophilic stress disrupts Keap1‐mediated ubiquitination of Nrf2, allowing Nrf2 to translocate into the nucleus and induce genes such as HO-1 and components of glutathione synthesis (Chu et al., 2024; Tanase et al., 2022). Activation of this pathway not only bolsters enzymatic ROS clearance but also inhibits iron‐driven lipid peroxidation, thereby directly countering ferroptosis (Jin and Chen, 2022). In DN, impaired Nrf2 nuclear translocation and reduced HO-1 expression exacerbate oxidative injury and ferroptotic susceptibility (Li et al., 2023a). By demonstrating that ACT decreases Keap1 levels, enhances Nrf2 nuclear accumulation, and upregulates HO-1, our data confirm that ACT harnesses this endogenous defense axis to suppress ferroptosis in DN (Figure 10).

Our study reveals a novel mechanism of ACT in DN, distinct from its traditionally recognized antioxidant and anti-inflammatory actions. Previous reports focused on ACT’s ability to attenuate oxidative stress and fibrosis through pathways like AKT/GSK-3β or autophagy-related regulators (Zhou et al., 2024). In contrast, we provide the first evidence that ACT ameliorates DN by targeting ferroptosis-driven cell death crucial in DN progression (Mengstie et al., 2023; Kim et al., 2021). Mechanistically, our findings suggest a functional interplay between the AGE/RAGE axis and the Keap1/Nrf2/HO-1 antioxidant pathway. Accumulation of AGEs and activation of RAGE are known to suppress Nrf2 signaling and elevate ROS production via NOX4 activation, thereby disturbing redox and iron homeostasis and priming cells for ferroptosis (Chen et al., 2024a; Ebrahimi et al., 2025). In parallel, Nrf2 not only promotes GPX4 expression but also regulates iron metabolism and lipid antioxidant defenses, mitigating ferroptotic vulnerability (Wang et al., 2023; Zhuo et al., 2022). Our data show that ACT downregulates AGE/RAGE signaling while enhancing Keap1/Nrf2/HO-1 activation, thus exerting dual protection: diminished upstream oxidative stress triggers and boosted downstream ferroptosis inhibitors. This coordinated mechanism establishes ACT as an innovative ferroptosis-targeting agent in DN therapy.

Collectively, these insights highlight the interdependent roles of oxidative stress and ferroptosis in DN pathogenesis and pinpoint the Keap1/Nrf2/HO-1 axis alongside its downstream effectors GPX4 and ACSL4 as a promising therapeutic nexus. In DN, AGE accumulation activates RAGE, which enhances ROS production via mitochondrial pathways, thereby promoting lipid peroxidation and iron overload that drive ferroptosis. GPX4 normally detoxifies lipid hydroperoxides to protect membrane integrity, while ACSL4 incorporation of polyunsaturated fatty acids sensitizes membranes to peroxidation. Dysregulation of these enzymes is both a marker and mediator of ferroptotic injury. Activation of Keap1/Nrf2/HO-1 not only upregulates antioxidant and iron-sequestration genes but also restores GPX4 function and limits ACSL4 activity, thereby breaking the vicious cycle of redox imbalance and ferroptosis. Therefore, it interrupts this pathogenic cycle at multiple nodes, highlighting its promise as a multifaceted ferroptosis-targeted therapy for DN.

Despite these compelling findings, several limitations must be acknowledged. First, while we have demonstrated the biochemical and histological benefits of ACT in an STZ-induced DN model, these results are preliminary and require further validation through additional experiments. Longer-term studies are needed to fully assess ACT’s safety profile, pharmacokinetics, and its capacity to forestall progression to renal fibrosis or end-stage renal disease.

Secondly, it should be noted that the docking results in this study are predictive rather than conclusive. While the molecular docking analysis suggests a potential interaction between ACT and key targets, further experimental validation, such as surface plasmon resonance or isothermal titration calorimetry, is required to confirm binding affinity and specificity.

Additionally, although we compared two ACT concentrations and observed that the high-dose (ACT-H) group consistently showed more pronounced therapeutic effects, including improved renal function, greater reduction of oxidative stress (e.g., MDA, LPO), and stronger modulation of ferroptosis-related markers (GPX4 upregulation, ACSL4 downregulation). We recognize that a more systematic dose-response evaluation is still lacking. Future studies should incorporate a wider dose range and pharmacodynamic profiling to better define the optimal therapeutic window and guide potential clinical translation.

## 5 Conclusion

Together, these findings reveal that ACT exerts multifaceted renoprotective effects in DN by intercepting the vicious cycle of mitochondrial dysfunction, oxidative stress, and ferroptosis through coordinated modulation of iron homeostasis and antioxidant pathways. This integrative systems-level approach not only advances our mechanistic understanding of ACT’s therapeutic potential but also underscores the promise of targeting ferroptosis and redox balance in the development of novel interventions for DN.

## Data Availability

The original contributions presented in the study are included in the article/Supplementary Material, further inquiries can be directed to the corresponding authors.

## Abbreviations

ACT, Acteoside; AGEs, advanced glycation end-products; AOD, average optical density; ACSL4, long-chain-fatty-acid-CoA ligase 4; BUN, blood urea nitrogen; DM, diabetes mellitus; DN, diabetic nephropathy; ESRD, end-stage renal disease; FBG, fasting blood sugar; GO, gene ontology; GSH, glutathione, r-glutamyl cyste1ingl + glycine; GPX4, glutathione peroxidase GPX4; H&E, hematoxylin-eosin; Keap1, Kelch-like ECH-associated protein 1; KEGG, Kyoto encyclopedia of genes and genomes; MDA, Malondialdehyde; Nrf2, nuclear factor erythroid 2-related factor 2; PASM, periodic acid-silver metheramine; PCR, polymerase chain reaction; PI3K, phosphatidylinositol 3 kinase; PPI, protein-protein interactions; ROS, reactive oxygen species; Scr, serum creatinine; SOD, superoxide dismutase; STZ, streptozotocin; TCM, traditional Chinese medicine; UPR, unfolded protein response; WB, western blot.
